# 

**Published:** 1843-02-18

**Authors:** 


### THE MEDICAL EXAMINER,

### And Retrospect of the Medical Sciences.

EDITED BY

MEREDITH CLYMER, M.D.

VOL. VI.] PHILADELPHIA, FEBRUARY 18, 1843. [No. 3.

